# Clearance of 1–2 cm Renal Stones After Subjecting the Patients to Extracorporeal Shockwave Lithotripsy With and Without Double J Ureteral Stent

**DOI:** 10.7759/cureus.95712

**Published:** 2025-10-29

**Authors:** Muhammad Khalid, Tariq Saleem Khan, Riaz Ahmad Khan, Ahmad Nawaz, Heraa Javed, Abdul Basit

**Affiliations:** 1 Department of Accident and Emergency, Mian Rashid Hussain Shaheed Memorial Hospital, Pabbi, PAK; 2 Department of Renal Transplantation, Institute of Kidney Diseases, Peshawar, PAK; 3 Department of Urology, Institute of Kidney Diseases, Peshawar, PAK; 4 Department of Radiology, MMC General Hospital, Peshawar, PAK

**Keywords:** kidney calculi, lithotripsy, postoperative complications, treatment outcome, ureteral stents

## Abstract

Introduction: Renal stones measuring 1-2 cm are a common urological problem frequently managed with extracorporeal shock wave lithotripsy (ESWL). However, the role of pre-procedural double J (DJ) stenting in improving stone clearance and minimizing complications remains controversial. Some studies suggest that stenting facilitates fragment passage and prevents steinstrasse, whereas others report increased patient discomfort and urinary symptoms.

Objective: The objective of this study was to evaluate whether pre-ESWL DJ stenting improves stone clearance and reduces procedure-related complications in patients with 1-2 cm renal stones.

Hypothesis: Pre-procedural DJ stenting facilitates fragment clearance and lowers obstruction-related events without significantly increasing overall morbidity.

Materials and methods: This was a prospective comparative study conducted in Pakistan from January 2022 to December 2024. A total of 230 patients with single renal stones (1-2 cm) were randomized into Group A (ESWL with pre-procedural DJ stenting) (n = 115) and Group B (ESWL without stenting) (n = 115). In Group A, 6 Fr polyurethane DJ stents were inserted under local or spinal anesthesia 24-48 hours before ESWL. Procedures were performed using lithotripters operated using standardized parameters (energy range 12-21 kV, up to 3,000 shocks per session, and 60-90 shocks per minute. A maximum of three sessions was performed, spaced 10-14 days apart. All patients received standardized analgesic (diclofenac sodium 75 mg IM) and prophylactic antibiotic (ciprofloxacin 500 mg twice daily for three days) regimens. Stents were removed after achieving clearance or within four weeks. Stone clearance was evaluated at two and four weeks post procedure using ultrasonography and non-contrast CT (NCCT) when needed. Complete clearance was defined as the absence of fragments >4 mm. Complications, including hematuria, renal colic, infection, steinstrasse, and stent-related symptoms, were recorded. Data were analyzed using chi-square and t-tests; multivariate logistic regression identified independent predictors of stone clearance.

Results: Complete clearance was achieved in 95 (82.6%) stented versus 78 (67.8%) non-stented patients (p = 0.012). Residual stones >4 mm occurred in five (4.3%) stented and 15 (13.0%) non-stented patients (p = 0.021). Hematuria was more frequent in the stent group (n=20; 17.4%) than in the non-stent group (n=10; 8.7%) (p = 0.048). Stent-related symptoms were reported in 28 (24.3%) patients. Steinstrasse occurred more commonly in the non-stent group (n=15; 13.0%) than the stent group (n=4; 3.5%) (p = 0.011), while renal colic was seen in 14 (12.2%) non-stented versus six (5.2%) stented patients (p = 0.049). Multivariate analysis confirmed DJ stenting as an independent predictor of clearance (OR = 2.14; 95%CI: 1.22-3.74; p = 0.007).

Conclusion: Pre-ESWL DJ stenting is associated with higher stone clearance and fewer obstructive complications, such as steinstrasse and colic, but increases stent-related morbidity. Routine use in all patients is not justified; selective stenting should be considered for cases at higher risk of obstruction, including larger or impacted stones, solitary kidneys, or unfavorable anatomical configurations.

## Introduction

Renal calculi, commonly referred to as kidney stones, represent a significant urological condition affecting individuals worldwide [1]. Their incidence has been increasing steadily, with prevalence rates ranging from 5% to 15% globally, and a higher recurrence risk among patients with a history of nephrolithiasis [2]. Stones measuring 1-2 cm are particularly challenging, often leading to pain, urinary obstruction, infection, and potential deterioration of renal function [3]. Effective management of these stones is essential to prevent complications and improve patient quality of life [4].

Among the various treatment modalities available, extracorporeal shock wave lithotripsy (ESWL) has emerged as a minimally invasive and widely adopted technique for the management of small to medium-sized renal stones [5]. ESWL works by delivering focused shock waves externally to fragment stones into smaller particles, which can then be naturally expelled through the urinary tract [6]. The procedure is safe, non-invasive, repeatable, and associated with shorter recovery times compared to conventional surgical interventions [7]. However, its success in achieving complete stone clearance can be influenced by several factors, including stone size, composition, location, patient anatomy, and the presence of urinary obstruction [8]. Reported success rates for stones measuring 1-2 cm range from 70% to 90%, but incomplete fragmentation or residual fragments remain a clinical challenge [5].

The use of double J (DJ) ureteral stents before ESWL has been proposed to facilitate drainage, prevent obstruction, and reduce post-procedural complications such as steinstrasse (stone street formation) [9]. Several studies have suggested that pre-stenting may enhance stone clearance by maintaining ureteral patency and reducing obstruction-related complications. In contrast, other studies have found no significant improvement in clearance rates and reported increased patient discomfort, urinary tract infections, and hematuria associated with stent use [10]. Consequently, the clinical benefit of routine pre-ESWL DJ stenting remains inconclusive, particularly for stones in the 1-2 cm range [11].

Given these considerations, a focused evaluation of ESWL outcomes with and without pre-procedural DJ stenting is warranted. Limited comparative data exist specifically for renal stones measuring 1-2 cm, where treatment decisions often remain empirical. Therefore, this study aims to evaluate the effect of pre-ESWL DJ stenting on stone clearance and procedure-related complications, addressing the existing uncertainty regarding its necessity and clinical effectiveness in patients undergoing ESWL for moderate-sized renal stones. The primary objective is to compare stone clearance rates between stented and non-stented groups, while the secondary objective is to assess procedure-related complications, including hematuria, infection, stent-related symptoms, and steinstrasse formation. It is hypothesized that pre-procedural DJ stenting enhances stone clearance and reduces obstructive complications but may increase stent-related morbidity.

## Materials and methods

Study design and setting

This comparative prospective study was conducted at the Institute of Kidney Diseases, Hayatabad Medical Complex, Peshawar, Pakistan, and the Department of Urology, Lady Reading Hospital Medical Teaching Institute, Peshawar, Pakistan. The study spanned a period of 36 months, from January 2022 to December 2024. The objective was to evaluate the effect of pre-ESWL DJ stenting on stone clearance and related complications in patients presenting with renal stones measuring 1-2 cm, confirmed by ultrasonography or non-contrast computed tomography (NCCT).

Sample size calculation

The sample size was estimated using the World Health Organization (WHO) formula for comparing two proportions [12]. The formula used was $n = \frac{(Z_{\alpha/2} + Z_{\beta})^2 \times [p_1(1 - p_1) + p_2(1 - p_2)]}{(p_1 - p_2)^2}$, Here, Z_α/2_ is the critical value of the standard normal distribution corresponding to a two-tailed significance level of 0.05 (Z_α/2_=1.96), representing a 95% confidence level, and Z_β_=0.84 corresponds to 80% power (β=0.20). The proportions p_1_ and p_2_ were derived from a previous study that reported stone-clearance rates of 66.7% for ESWL alone and 83.3% for ESWL with brief stenting in 1-2 cm stones [13]. Based on these values, the calculated sample size was 103 patients per group. To account for an anticipated 10% loss to follow-up, the final total sample size was increased to 230 patients, with 115 patients in each group.

Inclusion and exclusion criteria

Patients were eligible for inclusion if they were between 18 and 65 years of age, had a single renal stone measuring 1-2 cm in its largest diameter, and demonstrated normal renal function with serum creatinine within the reference range. Patients were excluded if they had multiple renal stones or stones larger than 2 cm, a history of previous urological surgery or intervention on the affected kidney, active urinary tract infection, bleeding disorders, pregnancy, or severe comorbidities that could affect treatment compliance or safety.

Randomization and blinding

Eligible participants were randomized using a computer-generated simple randomization list into two treatment groups. Group A underwent ESWL with pre-procedural DJ stenting, whereas Group B received ESWL without stent placement. Allocation concealment was maintained using sealed opaque envelopes. To minimize observer bias, the radiologist responsible for assessing stone clearance and complications remained blinded to the group assignments.

Pre-procedural evaluation

Each patient underwent a detailed clinical history and physical examination prior to the procedure. Baseline investigations included a complete blood count, serum creatinine, urinalysis, and urine culture. Imaging studies such as ultrasonography and NCCT were performed to determine stone size, location, and density. A single dose of intravenous ceftriaxone (1 g) was administered as antibiotic prophylaxis. Patients with positive urine cultures were treated with culture-specific antibiotics before ESWL. All participants provided written informed consent after the study objectives and procedures were explained to them.

Intervention

In Group A, a 5 Fr polyurethane DJ ureteral stent (24-26 cm in length) was inserted under local or spinal anesthesia 24 to 48 hours before ESWL. The ESWL procedure was carried out using a Dornier Compact Delta II lithotripter (Dornier MedTech, Munich, Germany) at the Institute of Kidney Diseases, Peshawar, and a Siemens Lithostar Modularis® lithotripter (Siemens Medical Solutions, Erlangen, Germany) at the Lady Reading Hospital, Peshawar. The difference in devices reflected the availability at each participating center.

Both lithotripters were operated using standardized parameters (energy range 12-21 kV, up to 3,000 shocks per session, and 60-90 shocks per minute). Each patient received up to three ESWL sessions at two-week intervals, depending on the stone fragmentation and clearance response.

Analgesia was provided with an intramuscular injection of diclofenac sodium (75 mg) prior to the procedure, followed by oral analgesics as required. Patients in Group B underwent ESWL under the same procedural conditions but without stent placement. The DJ stents were removed two to three weeks after the final ESWL session, or earlier if patients developed stent-related symptoms.

Follow-up and outcome assessment

Patients were followed up at two weeks and four weeks after ESWL. Ultrasonography was performed at each follow-up visit to assess residual fragments. In cases of suspected residual stones, NCCT was used for confirmation. Complete stone clearance was defined as the absence of visible fragments or the presence of clinically insignificant fragments measuring less than 4 mm [14,15]. Complications such as hematuria, urinary tract infection, renal colic, and stent-related symptoms were documented and graded using the Institutional Urological Complication Grading System (IUCGS), a structured scale locally developed by our urology research team specifically for this study. It was designed to categorize urological complications based on severity, required intervention, and impact on recovery.

Data analysis

All data were analyzed using IBM SPSS Statistics for Windows, version 25.0 (IBM Corp., Armonk, New York, United States). Continuous variables were expressed as mean ± standard deviation (SD), while categorical variables were presented as frequencies and percentages. The chi-square test was used to compare stone clearance rates between groups. Multivariate logistic regression analysis was applied to adjust for potential confounding variables such as stone size, stone location, and patient age. A p-value < 0.05 was considered statistically significant.

Ethical considerations

The study protocol was reviewed and approved by the Ethical Review Board of Lady Reading Hospital Medical Teaching Institute (approval number: 566/LRH/MTI, dated August 18, 2020) and the Hospital Research and Ethics Committee of Hayatabad Medical Complex (approval number: 814/CD/HMC/2023, dated December 18, 2023). Written informed consent was obtained from all participants before enrollment. Patient confidentiality was maintained throughout the study. Participation was voluntary, and patients retained the right to withdraw at any stage without affecting their clinical care.

## Results

A total of 230 patients were enrolled in the study, divided equally into two groups: ESWL with DJ stent (Group A, n=115) and ESWL without a DJ stent (Group B, n=115). As shown in Table 1, the mean age of Group A was 43.1 ± 12.0 years compared to 42.1 ± 11.6 years in Group B, with no significant difference (t=0.61, p=0.54). Gender distribution was also comparable, with 70 (60.9%) male and 45 (39.1%) female participants in Group A versus 68 (59.1%) male and 47 (40.9%) female participants in Group B (χ²=0.68, p=0.41). The mean stone size was 1.54 ± 0.27 cm in Group A and 1.51 ± 0.25 cm in Group B (t=0.69, p=0.49). Stone location (renal pelvis, calyx, ureter) was also similar between groups (χ²=0.24, p=0.88). BMI did not differ significantly, averaging 25.2 ± 3.1 kg/m² in Group A versus 24.9 ± 3.4 kg/m² in Group B (t=0.74, p=0.46).

**Table 1:** Baseline characteristics of study participants (N=230) Continuous variables (age, stone size, BMI) were compared using independent-sample t-tests, while categorical variables (gender, stone location) were analyzed using chi-square (χ²) tests. No significant differences were observed between groups for age (t = 0.61, p = 0.54), stone size (t = 0.69, p = 0.49), BMI (t = 0.74, p = 0.46), gender (χ² = 0.68, p = 0.41), or stone location (χ² = 0.24, p = 0.88). All p-values exceeded 0.05, indicating that both groups were comparable at baseline, confirming adequate randomization and control of potential confounding factors prior to outcome analysis.

Variable		Group A (n=115)	Group B (n=115)	Test statistic	p-value
Age (years), mean ± SD		43.1 ± 12.0	42.1 ± 11.6	t = 0.61	0.54
Gender, n (%)	Male	70 (60.9%)	68 (59.1%)	χ² = 0.68	0.41
	Female	45 (39.1%)	47 (40.9%)		
Stone Size (cm), mean ± SD		1.54 ± 0.27	1.51 ± 0.25	t = 0.69	0.49
Stone Location	Pelvis	50 (43.5%)	52 (45.2%)	χ² = 0.24	0.88
	Calyx	40 (34.8%)	38 (33.0%)		
	Ureter	25 (21.7%)	25 (21.7%)		
BMI (kg/m²), mean ± SD		25.2 ± 3.1	24.9 ± 3.4	t = 0.74	0.46

**Figure 1:**
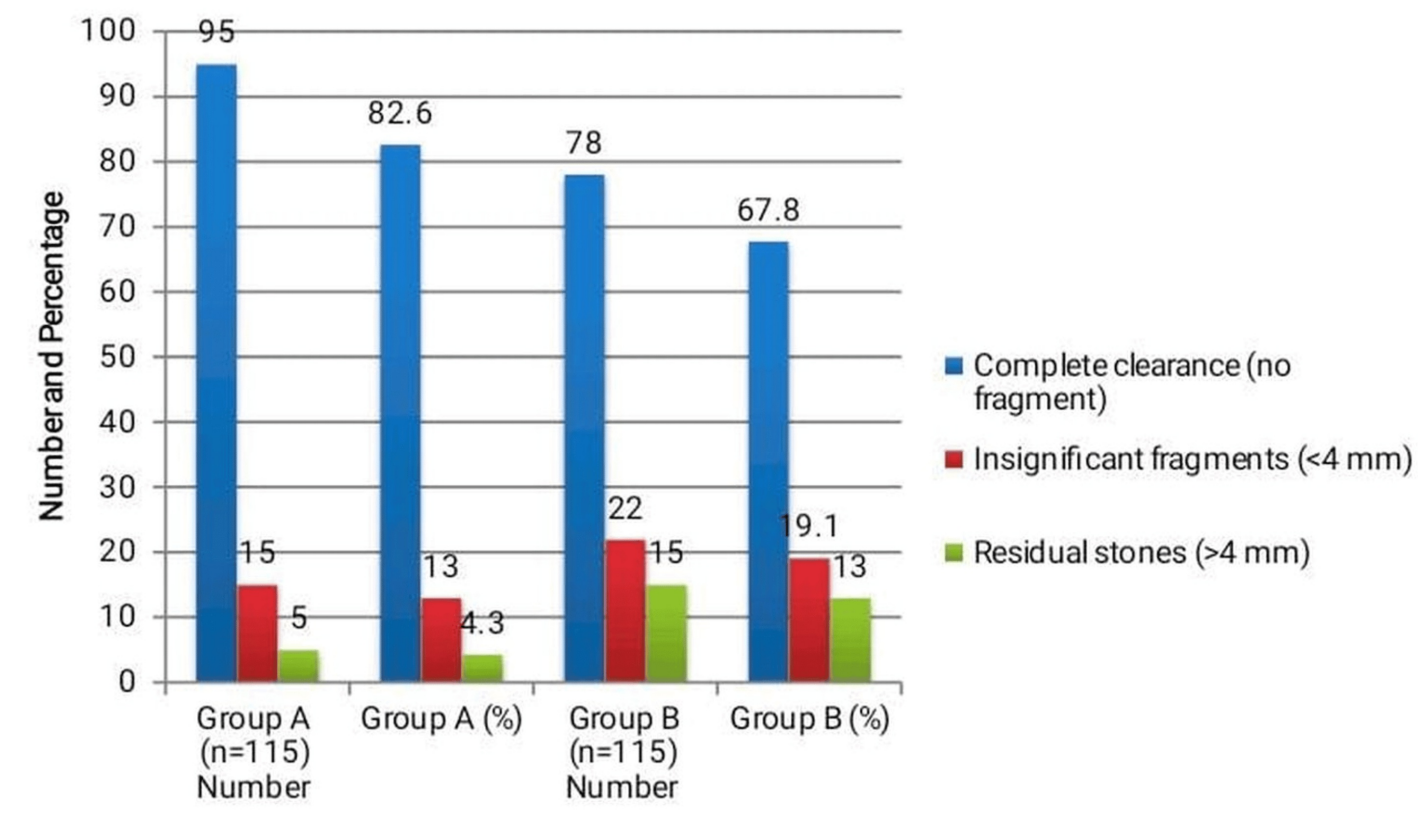
Stone clearance outcomes Comparison of stone clearance between ESWL with and without DJ stenting was performed using chi-square (χ²) tests. Complete clearance was significantly higher in the stented group (χ² = 6.33, p = 0.012), while residual stones > 4 mm were more frequent in the non-stented group (χ² = 5.28, p = 0.021). The difference in the rate of insignificant fragments < 4 mm was not statistically significant (χ² = 1.58, p = 0.21). After adjusting for potential confounders including stone size, location, and age in multivariate analysis, DJ stenting remained an independent predictor of complete stone clearance.

Stone clearance was significantly higher in Group A compared to Group B. As illustrated in Figure 1, in Group A, 95 (82.6%) patients achieved complete clearance vis-à-vis 78 (67.8%) patients in Group B (χ²=6.33, p=0.012). Residual stones (>4 mm) were observed in five (4.3%) patients in Group A compared to 15 (13.0%) in Group B (χ²=5.28, p=0.021). Insignificant fragments (<4 mm) were present in 15 (13.0%) patients in Group A and 22 (19.1%) in Group B, but this difference was not statistically significant (χ²=1.58, p=0.21). These findings indicate a clear benefit of DJ stenting for stone clearance.

Complication rates varied significantly between groups. As shown in Table 2, hematuria occurred in 20 (17.4%) patients in Group A versus 10 (8.7%) patients in Group B (χ²=3.89, p=0.048). Steinstrasse was significantly more common in Group B (n=15; 13.0%) patients, compared to Group A (n=4; 3.5%) (χ²=6.47, p=0.011). Renal colic was also more frequent in Group B (n=14; 12.2%) versus Group A (n=6; 5.2%) (χ²=3.88, p=0.049). Stent-related symptoms, however, were noted only in Group A (n=28; 24.3%). No significant difference was observed for UTI rates (n=12 (10.4%) vs. n=8 (7.0%); χ²=0.84, p=0.36).

**Table 2:** Post-procedural complications after ESWL with and without DJ stenting, classified according to the IUCGS developed and used at our centers for procedural audits. Grade I: minor/self-limiting (e.g., mild hematuria, transient pain); Grade II: requiring medical therapy (e.g., UTI, stent discomfort); Grade III: requiring procedural intervention (e.g., steinstrasse requiring ureteroscopy); Grade IV: life-threatening (e.g., sepsis, renal failure); Grade V: death related to the procedure. Chi-square analysis showed significant differences for hematuria (p=0.048), steinstrasse (p=0.011), and renal colic (p=0.049), favoring the stented group. No Grade IV–V events occurred. Confounding variables (stone size, location, BMI, and age) were adjusted for in multivariate analysis, confirming DJ stenting as an independent predictor of improved clearance with acceptable morbidity. ESWL: extracorporeal shock wave lithotripsy; DJ: double J; IUCGS: Institutional Urological Complication Grading System

Complication	Group A (n=115)	Group B (n=115)	IUCGS Grade	χ² value	p-value
Hematuria	20 (17.4%)	10 (8.7%)	Grade I	χ² = 3.89	0.048*
UTI	12 (10.4%)	8 (7.0%)	Grade II	χ² = 0.84	0.36
Steinstrasse	4 (3.5%)	15 (13.0%)	Grade III	χ² = 6.47	0.011*
Renal colic	6 (5.2%)	14 (12.2%)	Grade II–III	χ² = 3.88	0.049*
Stent-related symptoms	28 (24.3%)	–	Grade II	–	–

**Figure 2:**
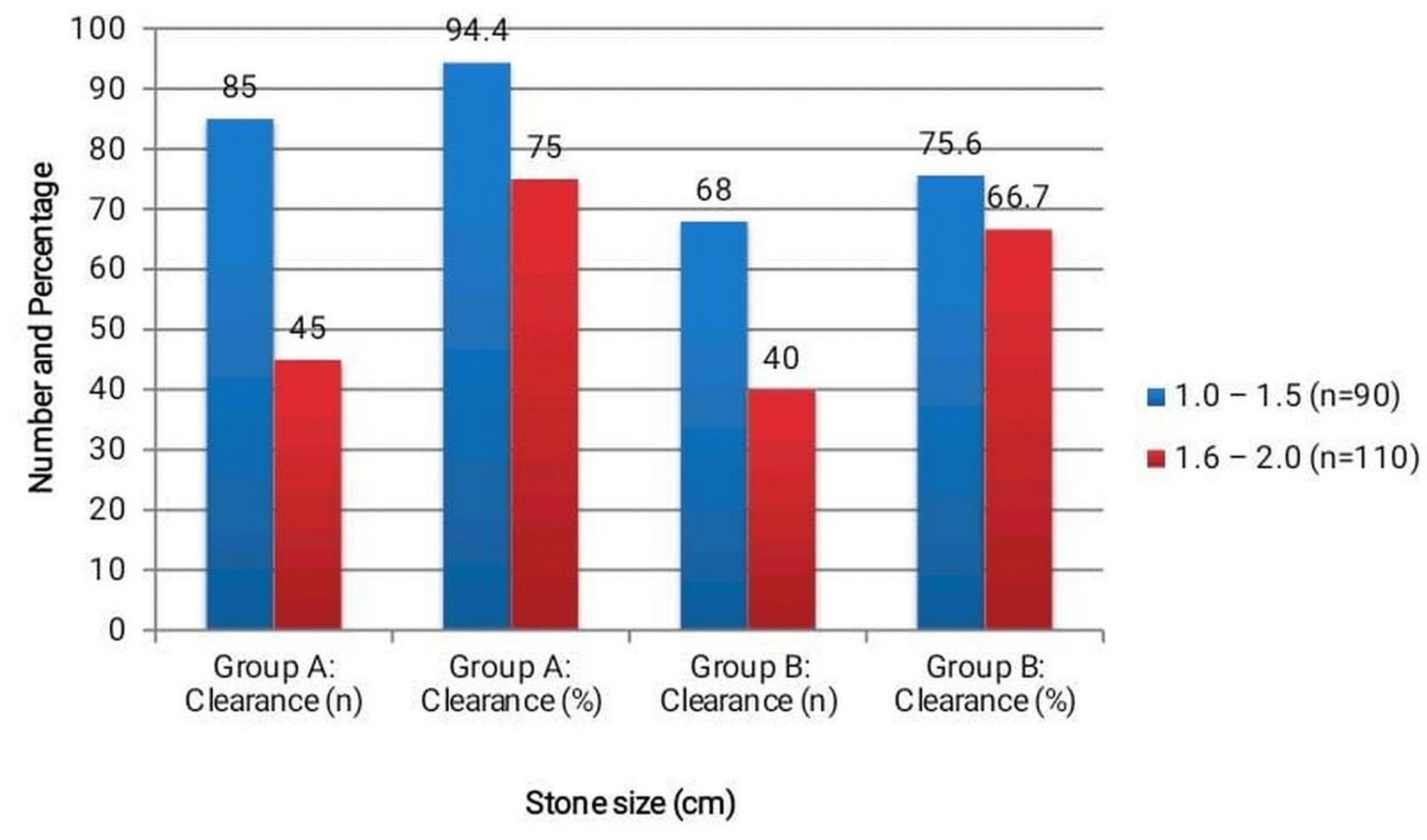
Stone clearance rates were analyzed according to stone size categories (1.0–1.5 cm and 1.6–2.0 cm) in patients undergoing ESWL with and without DJ stenting. For stones measuring 1.0–1.5 cm, clearance was significantly higher in the stented group (85/90) compared to the non-stented group (68/90) (χ² = 10.95, p = 0.001). In contrast, for stones measuring 1.6–2.0 cm, the difference between groups (45/60 vs. 40/50) was not statistically significant (χ² = 1.20, p = 0.27). Multivariate logistic regression confirmed that the advantage of DJ stenting for smaller stones (1.0–1.5 cm) remained significant after adjusting for potential confounders such as age and stone location. ESWL: extracorporeal shock wave lithotripsy; DJ: double J

When stratified by stone size, clearance rates were significantly higher for smaller stones in Group A. For stones measuring 1.0-1.5 cm, clearance was achieved in 85 (94.4%) patients in Group A compared to 68 (75.6%) patients in Group B (χ²=10.95, p=0.001). As illustrated in Figure 2, for larger stones measuring 1.6-2.0 cm, clearance was observed in 45 (75.0%) patients in Group A versus 40 (66.7%) patients in Group B (χ²=1.20, p=0.27), which was not statistically significant. These findings suggest that DJ stenting is particularly effective for smaller stones.

Multivariate analysis identified DJ stenting as a significant independent predictor of stone clearance. As shown in Table 3, patients with DJ stents had more than two-fold higher odds of clearance (OR=2.14, 95%CI: 1.22-3.74, Wald χ²=7.27, p=0.007). Stone size greater than 1.5 cm was associated with lower clearance (OR=0.61, 95%CI: 0.35-1.08, p=0.09), though not statistically significant. Neither stone location (OR=1.28, p=0.39) nor age above 45 years (OR=0.87, p=0.64) was an independent predictor. This confirms that DJ stenting provides a significant therapeutic advantage after adjusting for confounders.

**Table 3:** Multivariate logistic regression analysis for predictors of stone clearance (confounder-adjusted model) Multivariate logistic regression analysis showing predictors of stone clearance after ESWL, controlling for potential confounders (stone size, location, age, gender, and BMI). DJ stent use remained a significant independent predictor of successful stone clearance (OR = 2.14, 95% CI 1.22–3.74, p = 0.007). Other variables were not significant. The model demonstrated acceptable fit (Hosmer–Lemeshow p = 0.64) and explained 18.2% of variance (Nagelkerke R² = 0.182). *statistically significant (p < 0.05) — meaning the association between DJ stent use and stone clearance is unlikely to be due to chance. DJ: double J

Variable	Adjusted OR	95% CI	Wald χ²	p-value
DJ stent use	2.14	1.22 – 3.74	7.27	0.007*
Stone size (>1.5 cm)	0.61	0.35 – 1.08	2.88	0.09
Stone location (pelvis vs. calyx/ureter)	1.28	0.72 – 2.28	0.73	0.39
Age (>45 years)	0.87	0.49 – 1.53	0.22	0.64
BMI (>25 kg/m²)	0.93	0.54 – 1.59	0.09	0.76
Gender (male)	1.12	0.64 – 1.96	0.13	0.72

## Discussion

This study demonstrated that patients who underwent ESWL with pre-procedural DJ stenting had higher stone-free rates compared to those without stents, particularly for stones measuring 1-1.5 cm. Stented patients also experienced fewer obstructive complications, such as steinstrasse and renal colic. However, the use of DJ stents was associated with higher rates of stent-related irritative symptoms and hematuria. After adjusting for stone size, location, and patient age, stenting remained an independent predictor of stone clearance. Overall, the findings suggest that DJ stenting improves clearance outcomes but introduces additional morbidity that must be weighed clinically.

Several clinical studies that evaluated brief pre-stenting before ESWL for 1-2 cm renal stones have reported higher immediate clearance rates and fewer repeat sessions, consistent with our findings [13]. Our results also support the observation that stenting reduces the risk of clinically significant residual fragments and the development of steinstrasse [16].

On the other hand, multiple randomized controlled trials and meta-analyses have argued that routine pre-stenting does not consistently improve stone-free rates and instead leads to more urinary symptoms and discomfort [17]. This aligns with our study’s finding that while clearance rates were better with stenting, a considerable proportion of patients (about one-fourth) reported stent-related morbidity [18].

With respect to obstructive complications, prior studies have highlighted that stenting offers protection against fragment aggregation and obstruction, particularly for larger stones or those in challenging anatomical positions [19]. Similarly, our study showed a significant reduction in steinstrasse and colic in the stented group, confirming this protective effect [20].

Stone size and location have been widely recognized as major determinants of ESWL success [21]. The current findings demonstrated a significant advantage of stenting for smaller stones (1-1.5 cm), whereas the benefit was less pronounced for larger stones (1.6-2.0 cm) [22]. This observation mirrors earlier research that emphasized the heterogeneity of ESWL outcomes and suggested selective stenting in particular subgroups rather than a routine approach [23].

Taken together, the results from this study and prior literature suggest that while DJ stenting may not be necessary for all patients undergoing ESWL, it can offer significant benefits in selected cases, especially for smaller stones or where obstruction risk is high [10]. However, this must be balanced against the discomfort and morbidity associated with stent placement [24].

Limitations and future suggestions

This study was limited by its single-center design, short follow-up period, and reliance on ultrasound rather than CT for detecting residual fragments. Important variables such as stone density, shock parameters, and validated patient-reported outcomes were not assessed, which may have influenced the findings. Future studies should be multicenter with longer follow-up, include detailed stone and procedural characteristics, and utilize standardized symptom scores. Research on newer stent designs and predictive models may further guide selective rather than routine use of DJ stenting before ESWL.

## Conclusions

The present study demonstrates that pre-procedural DJ stenting offers a measurable benefit in enhancing stone clearance rates and minimizing post-ESWL obstructive complications in patients with renal calculi measuring 1-2 cm. Patients who underwent stenting prior to ESWL experienced smoother fragment passage and fewer obstruction-related issues, supporting its role in improving procedural safety and efficacy. However, these advantages were offset by a notable increase in stent-related discomfort and morbidity, including urinary symptoms and the need for additional interventions. Therefore, routine pre-stenting for all patients is not justified. Instead, DJ stenting should be reserved for selective cases, particularly those with large, impacted stones, solitary kidneys, or anatomical factors predisposing to obstruction, where the clinical benefits outweigh potential drawbacks. 
